# Chicago Medico-Historical Society

**Published:** 1874-09-01

**Authors:** 


### CHICAGO MEDICO-HISTORICAL SOCIETY.

A SPECIAL meeting of this Society was held in the parlor of the Tremont House, Thursday evening, August 20th. The Secretary stated that on the Monday evening previous he had mailed to each member of the Society a proof sheet containing the list of regular physicians in the city of Chicago, as passed upon by the Society at its previous meetings. This was done in order that members who had not been present at all of the previous meetings, might have an opportunity to scrutinize the entire list, and, at the present meeting, to call for a reconsideration of any names to which they might take exception.

Unfortunately, however, by some fault of the Post-office, these lists reached the members only on Wednesday evening, or during the following day, Thursday, a few hours previous to this meeting called to finally consider them.

It was suggested, therefore, that the final action on the list be postponed for one week, in order that the names might be more thoroughly canvassed and the necessary facts and evidence obtained regarding the doubtful cases. This proposition was, however, overruled by the Society.

Several names previously missed or passed over for want of information, were brought up, and being favorably endorsed, were passed for admission to the list.

The vote of admission was also reconsidered in quite a number of instances, and the standing of the parties more fully canvassed. The names of two or three of the younger *wrong-doers*, poor, friendless, and with nobody to advocate their cause, were expunged.

The most objectionable names on the list, however, including several open, acknowledged and universally recognized advertising quacks and charlatans, were either not called up at all, or, when brought forward, were defended by the eloquent appeals of a certain clique who privately favor and sustain, although they have not the courage to openly advocate, the irregular and unprofessional practices of these men.

We fully expressed our opinions in the last number of the EXAMINER regarding this so-called list of regular physicians, which has been passed upon and endorsed by the Chicago Medico-Historical Society.

The attempt at this last meeting to revise and correct the list, proved an utter failure, and, as now ordered for publication, it stands essentially the same as when previously criticised by us. A more just or reasonable action is scarcely to be expected from this Society as at present constituted and officered.

Under the circumstances there is but one honorable course left for the respectable regular members of this Society to pursue; that is, to withdraw at once their names and influence, and thus allow the irregulars and quacks, and those who lower themselves to their level by advocating their recognition, to stand responsible for their own action, in the publishing of this *Register and Directory*.

